# Climate, Deer, Rodents, and Acorns as Determinants of Variation in Lyme-Disease Risk

**DOI:** 10.1371/journal.pbio.0040145

**Published:** 2006-05-09

**Authors:** Richard S Ostfeld, Charles D Canham, Kelly Oggenfuss, Raymond J Winchcombe, Felicia Keesing

**Affiliations:** **1**Institute of Ecosystem Studies, Millbrook, New York, United States of America; **2**Department of Biology, Bard College, Annandale-on-Hudson, New York, United States of America; Princeton UniversityUnited States of America

## Abstract

Risk of human exposure to vector-borne zoonotic pathogens is a function of the abundance and infection prevalence of vectors. We assessed the determinants of Lyme-disease risk (density and Borrelia burgdorferi-infection prevalence of nymphal Ixodes scapularis ticks) over 13 y on several field plots within eastern deciduous forests in the epicenter of US Lyme disease (Dutchess County, New York). We used a model comparison approach to simultaneously test the importance of ambient growing-season temperature, precipitation, two indices of deer *(Odocoileus virginianus)* abundance, and densities of white-footed mice *(Peromyscus leucopus),* eastern chipmunks *(Tamias striatus),* and acorns ( Quercus spp.), in both simple and multiple regression models, in predicting entomological risk. Indices of deer abundance had no predictive power, and precipitation in the current year and temperature in the prior year had only weak effects on entomological risk. The strongest predictors of a current year's risk were the prior year's abundance of mice and chipmunks and abundance of acorns 2 y previously. In no case did inclusion of deer or climate variables improve the predictive power of models based on rodents, acorns, or both. We conclude that interannual variation in entomological risk of exposure to Lyme disease is correlated positively with prior abundance of key hosts for the immature stages of the tick vector and with critical food resources for those hosts.

## Abstract 2

A long-term study of tick dynamics simultaneously assesses the impact of multiple ecological variables on Lyme disease risk and strongly implicates a role for rodent hosts and their food resources.

## Introduction

Many emerging and re-emerging infectious diseases of humans are zoonoses transmitted by vectors. Examples include West Nile virus, ehrlichiosis, anaplasmosis, tick-borne encephalitis and Lyme disease. In each case, the vector—usually a mosquito or tick—acquires the pathogen from a vertebrate host during a blood meal taken early in the life cycle and becomes capable of transmitting it to humans during a later blood meal. Risk of human exposure to the disease increases with increasing abundance and infection prevalence of the vectors [ 1, 2]. For virtually all vector-borne zoonoses, disease incidence in humans varies substantially from year to year [ 3– 5]. Determining the causes of interannual variation in entomological risk would facilitate the development and deployment of preventative measures, potentially reducing the burden of disease.

Lyme disease is the most frequently reported vector-borne disease in the US [ 6]. Lyme disease is most prevalent in northeastern and north-central regions where suburban and exurban development encroaches on deciduous forest ecosystems that support the pathogen, vector, and their vertebrate hosts [ 7]. The etiological agent is a spirochete bacterium, *Borrelia burgdorferi,* which is transmitted by ticks in the Ixodes ricinus complex. In the eastern and central US, the vector is the blacklegged tick, *Ixodes scapularis. I. scapularis* is a three-host tick, requiring three blood meals, one each as a larva, nymph, and adult, to fulfill its life cycle. Larval ticks hatch in midsummer, typically uninfected with *B. burgdorferi,* and begin seeking a host for their initial blood meal. After feeding on a vertebrate host for several days, the larvae drop off the host and molt into the nymphal stage, which undergoes diapause for almost a year before becoming active and seeking a host the following late spring or early summer. Both larvae and nymphs are highly nonspecific in their choice of hosts, parasitizing dozens of species of mammal, bird, and lizard [ 8]. After feeding to repletion, nymphs drop off the host and molt into the adult stage, which seeks a medium- or large-mammal host in midautumn of the same year. Infection with B. burgdorferi can be acquired from the host during either the larval or nymphal blood meal, and both nymphs and adults are capable of transmitting infection to a vertebrate host, including humans.

Owing to its tiny size, potentially high abundance, and summer feeding, the nymphal stage is most likely to transmit B. burgdorferi to people and hence is responsible for the great majority of Lyme-disease cases [ 9]. Risk of human exposure to Lyme disease, given entry into habitats where ticks occur (mainly forests; [ 10]), is a function of the density of infected nymphal ticks, which in turn is the product of the total density of nymphs (DON) and the nymphal infection prevalence (NIP) [ 11]. Determining the causes of variation in density of infected nymphs (DIN) and its component parts is an important goal with both ecological and epidemiological implications. Human behavioral patterns, such as time spent in forest habitat and protective measures taken against exposure to ticks, also influence Lyme-disease risk but are beyond the scope of this study.

Prior studies of the factors influencing DIN and DON have focused largely on variation in climate and in the abundance and distribution of white-tailed deer *(Odocoileus virginianus).* Because ticks spend greater than 95% of their lives on the forest floor either digesting the blood meal, undergoing diapause, or seeking a host, exposure to ambient temperature and humidity could be important to survival rates and population dynamics [ 12– 14]. In the laboratory, ticks experience high mortality when exposed to low humidity and high temperatures [ 15]. Consequently, hot and/or dry springs and summers have been postulated to reduce subsequent nymphal tick densities and Lyme-disease risk [ 16– 18].

Because adult I. scapularis feed predominantly on white-tailed deer [ 19], much research has evaluated the impact of variation in abundance of deer on abundance of ticks. When deer are eliminated from some habitats by hunting or fencing, the abundance of ticks typically is strongly reduced [ 20– 22]. Studies comparing natural variation in deer abundance with that in tick abundance are less conclusive; some have shown strong associations [ 23– 25], whereas others have not [ 26– 28].

Less attention has been paid to the potential effects of variation in abundance of hosts for larval ticks in influencing variation in DIN, DON, and NIP. This is perhaps a consequence of the lack of specialization by larvae on any particular host species. However, larval I. scapularis feed abundantly on white-footed mice *(Peromyscus leucopus),* and this host is the most competent natural reservoir for B. burgdorferi [ 29, 30]. High feeding success on mice combined with high reservoir competence has led some researchers to postulate that Lyme-disease risk will vary with mouse abundance [ 31, 32]. Although eastern chipmunks *(Tamias striatus)* host many larval ticks and are competent B. burgdorferi reservoirs [ 33], the impacts of variation in chipmunk abundance on Lyme-disease risk have been even less thoroughly explored (but see [ 34, 35]).

The food resources for tick hosts might also be important to Lyme-disease risk. Oak trees ( Quercus spp.) that dominate many forests in the US Lyme-endemic zone are known to produce highly variable acorn crops, a phenomenon known as masting. Acorns comprise a crucial resource for several vertebrate species, including white-footed mice, eastern chipmunks, and white-tailed deer, and can influence population density of the rodents [ 32, 36– 38] as well as space use by deer [ 39]. Jones et al. [ 40] described a dramatic increase in abundance of larval I. scapularis following experimental simulation of a masting event, but the scale of the experiments was insufficient to assess longer-term impacts on abundance of nymphs. In a follow-up, Ostfeld et al. [ 41] described a positive correlation between metrics of Lyme-disease risk and both prior-year mouse abundance and acorn abundance 2 y previously.

**Figure 1:**
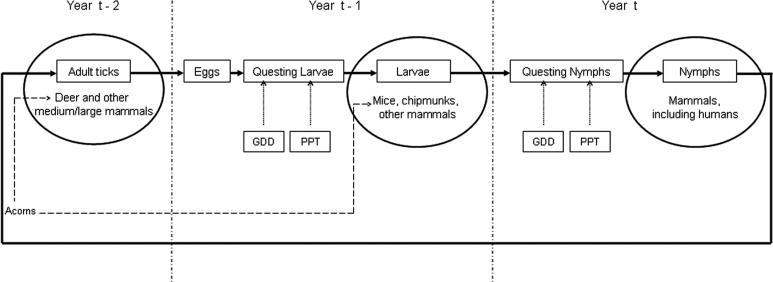
Diagram of Life Cycle of the Blacklegged Tick *(I. scapularis)* Shows the four life stages, egg, larva, nymph, adult, and the times during the life cycle that both abiotic (GDD, PPT), and biotic (acorns and various hosts) factors might exert influence. Year *t* is the year during which nymphal ticks seek hosts, including humans, and represents the focal year with respect to risk of exposure.

No prior study has assessed simultaneously the effects of variation in temperature, precipitation, deer, mice, chipmunks, and acorns, on variation in entomological risk of exposure to Lyme disease ( Figure 1). Assessments of subsets of these putative causal variables are characterized by relatively short time series, which have limited power to assess the influence of each factor separate from the others. Here we use long-term monitoring of these parameters combined with model comparison approaches to address the causes of variable risk in an area of high Lyme-disease incidence.

## Results

The principal entomological Lyme-disease risk factor, DIN, varied by an order of magnitude among years, ranging from 1.07 infected nymphs × 100 m ^−2^ in 1997 (averaged across all six plots) to 10.00 × 100 m ^−2^ in 1996. This variation was due primarily to variation in DON, which varied 6-fold among years (3.59 to 21.07 × 100 m ^−2^). In contrast, NIP varied less than 2-fold among years, from 0.24 in 2005 to 0.45 in 1999.

### Models for Total DON

**Figure 2:**
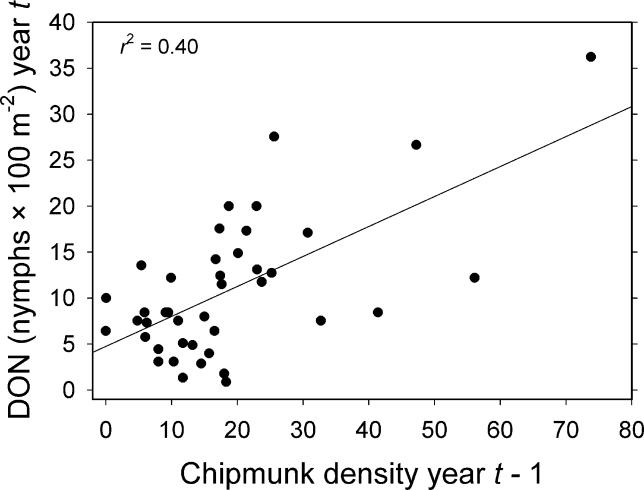
Effects of Population Density of Eastern Chipmunks *(T. striatus)* on DON Shows relationship between number of chipmunks per 2.25-ha grid in year *t*−1 and DON (number per 100 m ^2^) in year *t.* This regression model for DON had the most support.

Effect of growing degree days in the previous year (GDD *_t_*_−1_) on DON in the current year was positive but very weak in both the magnitude (estimated slope of the regression) and the strength of evidence for the effect ( Table 1). Total growing season precipitation in the current year (PPT *_t_*) also weakly influenced DON, but the effect was Gaussian with a peak at 223 mm. A model in which these two weather terms are combined multiplicatively was stronger than either of the univariate models, but explained only 15% of the variance ( Table 1). None of the other climate variables, deer variables, or prior year's density of larvae (DOL *_t_*_−1_) had any effect on DON; i.e., Akaike's information criterion corrected for small sample size (AIC _corr_) values were much higher than those of the means model. Abundance of acorns *_t_*_−2_, mice *_t_*_−1_, chipmunks *_t_*_−1_, and rodents *_t_*_−1_ (sum of mice and chipmunks) all independently influenced DON, and in all cases linear models were superior to exponential or power functions. The best univariate model for DON was a simple linear model of chipmunks *_t_*_−1_, which explained 40% of the variance ( Table 2; Figure 2). In no case was a multiple regression model superior to the chipmunk model ( Table 2).

**Table 1:**
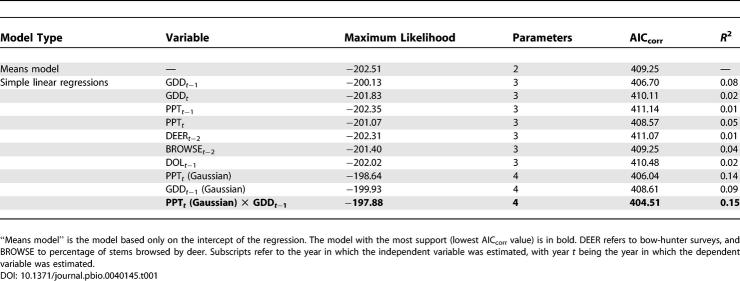
Model Comparison Statistics for Independent Variables Potentially Influencing the DON Based on the Full Dataset (58 Plot Years)

**Table 2:**
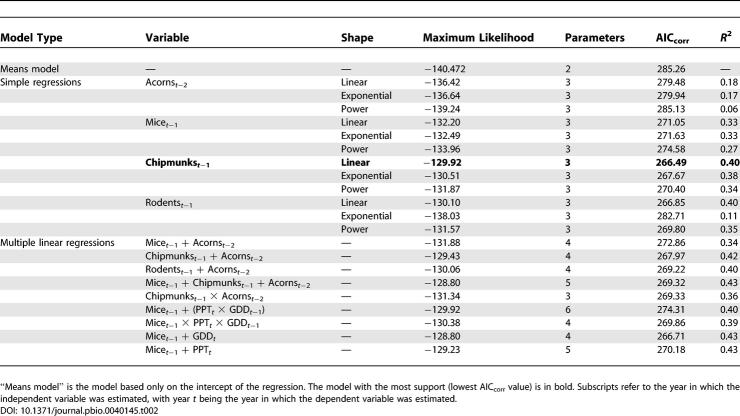
Model Comparison Statistics for Independent Variables Potentially Influencing the DON Based on the Subset of Plot Years for Which All Independent Variables Were Estimated (42 Plot Years)

### Models for NIP

**Figure 3:**
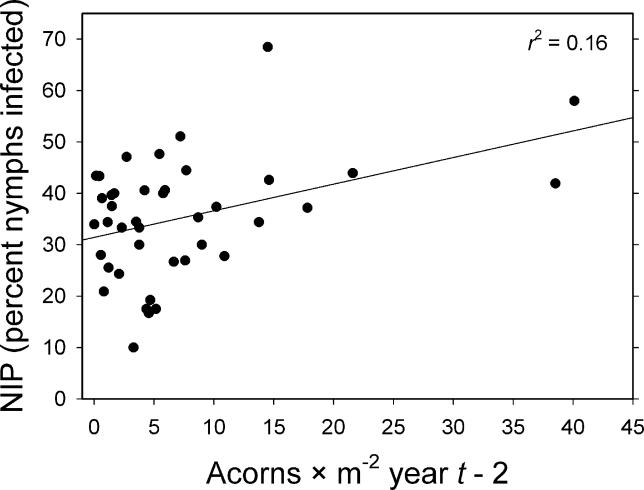
Effects of Acorn ( Quercus spp.) Density on NIP Shows effects of acorns per square meter in year *t*−2 on NIP (percentage of nymphs infected with B. burgdorferi) in year *t.* This regression model for NIP had the most support.

Among all univariate models, NIP responded only to the density of acorns in year *t*−2 ( Figure 3), and this relationship explained only 16% of the variance in NIP. Models incorporating the climate variables, deer variables, DOL, mice, chipmunks, and total rodents performed no better than the means model ( Tables 3 and 4). Because none of the independent variables other than acorns *_t_*_−2_ produced an improvement over the means model, there was no justification for testing multiple regression models.

**Table 3:**
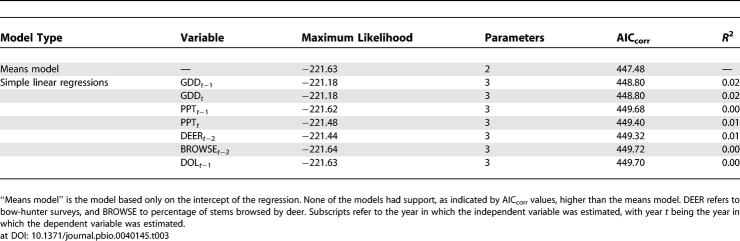
Model Comparison Statistics for Independent Variables Potentially Influencing NIP Based on the Full Dataset (58 Plot Years)

**Table 4:**
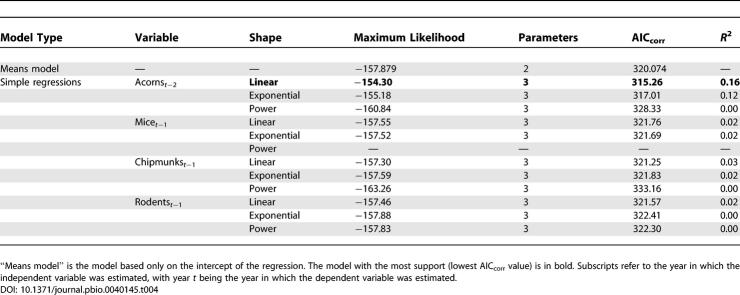
Model Comparison Statistics for Independent Variables Potentially Influencing NIP Based on the Subset of Plot-Years for Which All Independent Variables Were Estimated (42 Plot Years)

### Models for DIN

Similar to the results for DON, the effect of GDD *_t_*_−1_ on DIN in year *t* was positive but very weak in both the magnitude and the strength of evidence for the effect ( Table 5). Total PPT *_t_* also weakly influenced DIN, and again the effect was Gaussian with a peak at 213 mm of rainfall. A model in which these two weather terms are combined multiplicatively was stronger than either of the univariate models, but explained only 11% of the variance ( Table 5). None of the other weather variables, deer variables, nor DOL *_t_*_−1_ had any effect on DIN, with AIC _corr_ values higher than those of the means model. Abundance of acorns *_t_*_−2_, mice *_t_*_−1_, chipmunks *_t_*_−1_, and rodents *_t_*_−1_ all independently influenced DIN. For acorns *_t_*_−2_, mice *_t_*_−1_, and rodents *_t_*_−1_, the best models were nonlinear, but the difference in AIC _corr_ (ΔAIC _corr_) between the best model and corresponding linear model was always less than 1 (i.e., they had equivalent levels of support in the data), and the shapes of all nonlinear models were very close to linear ( Table 5). Consequently, we tested linear combinations of these predictor variables in multiple regressions.

**Table 5:**
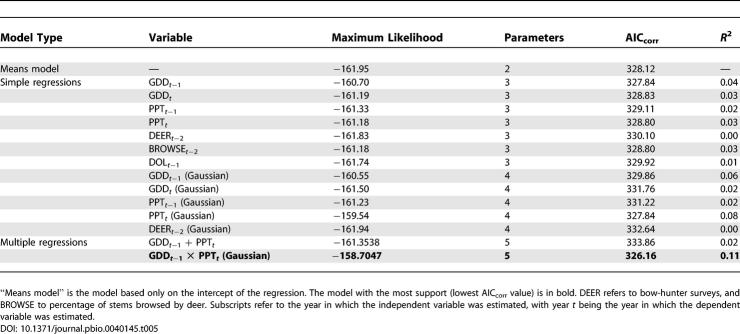
Model Comparison Statistics for Independent Variables Potentially Influencing the DIN Based on the Full Dataset (58 Plot Years)

**Figure 4:**
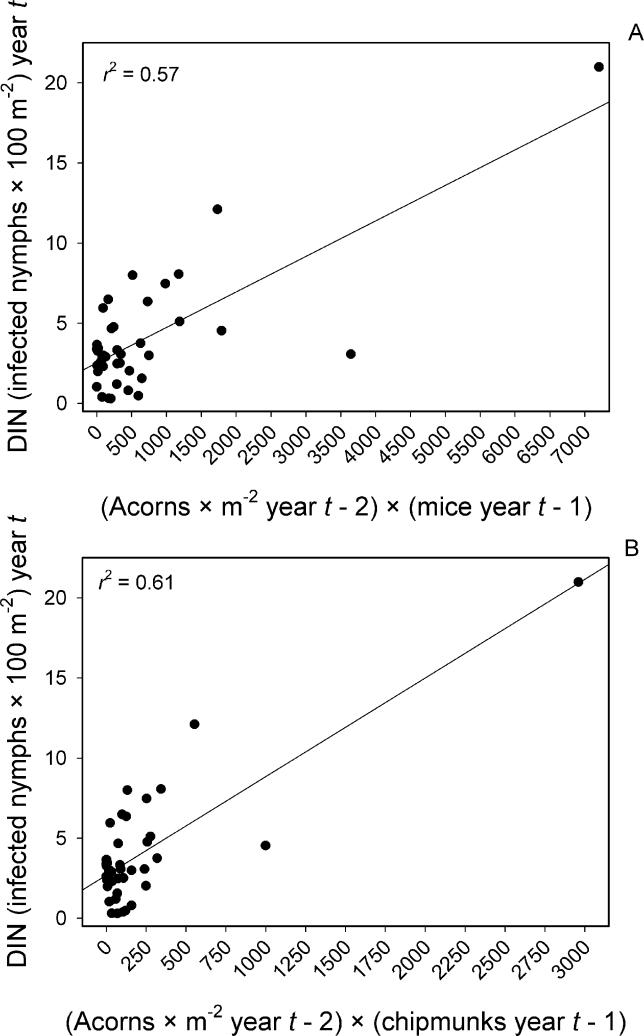
Effects of Acorn and Rodent Densities on DIN (A) Effects of the product of acorn density (acorns per square meter) in year *t*−2 and mouse *(P. leucopus)* density (number per 2.25-ha grid) in year *t*−1 on the density of B. burgdorferi-infected nymphs (number per 100 m ^2^) in year *t.* This regression model for DIN had the most support. (B) Effects of the product of acorn density (acorns per square meter) in year *t*−2 and chipmunk *(T. striatus)* density (number per 2.25-ha grid) in year *t*−1 on the density of B. burgdorferi-infected nymphs (number per 100 m ^2^) in year *t.* This regression model for DIN had nearly as much support (AIC _corr_) as the mouse model (A) and a higher *r* ^2^ value.

A model combining mice *_t_*_−1_ and acorns *_t_*_−2_ multiplicatively was the best model of all multiple regressions combining predictor variables that were supported by univariate analyses, and it explained 57% of the variance in DIN ( Table 6; Figure 4A). The model with multiplicative effects of chipmunks *_t_*_−1_ and acorns *_t_*_−2_ was nearly as good, with ΔAIC _corr_ = 1.05, and a slightly higher *R* ^2^ (0.61) ( Figure 4B).

**Table 6:**
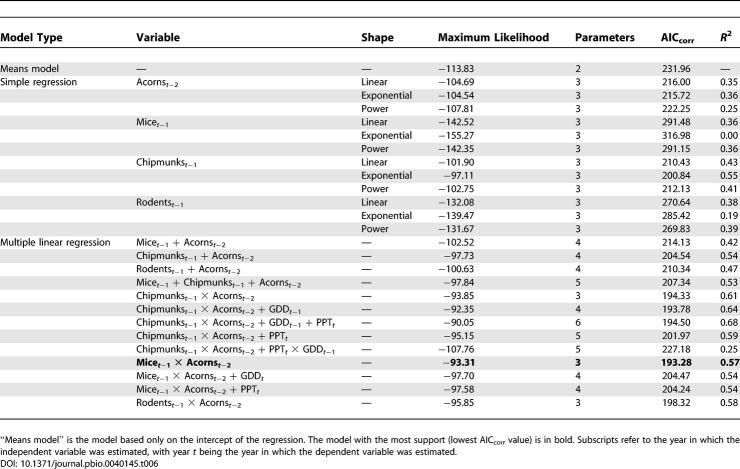
Model Comparison Statistics for Independent Variables Potentially Influencing the DIN Based on the Subset of Plot Years for Which All Independent Variables Were Estimated (42 Plot Years)

### Host Responses to Acorns

**Figure 5:**
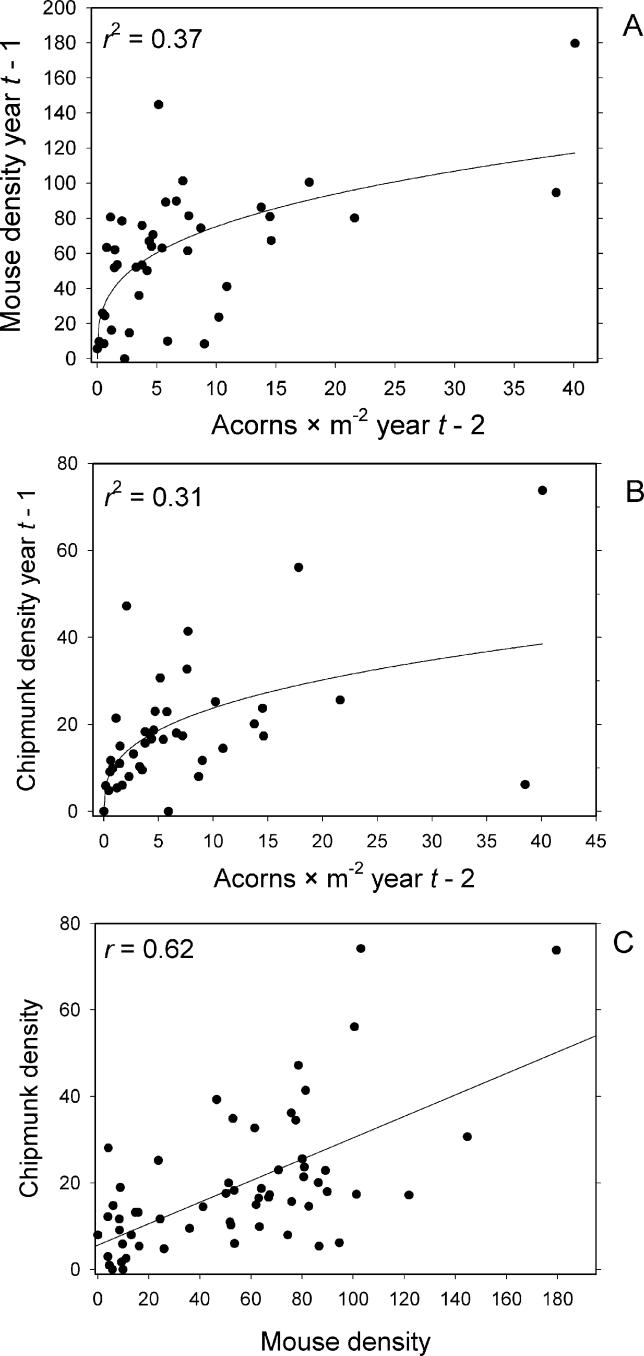
Effects of Acorn Density on Mouse and Chipmunk Densities Shows effects of acorn density (acorns per square meter) in year *t*−2 on (A) mouse *(P. leucopus)* density (number per 2.25-ha grid) in year *t*−1 and (B) chipmunk *(T. striatus)* density (number per 2.25-ha grid) in year *t*−1. (C) Correlation between mouse density and chipmunk density across plots and years.

**Figure 6:**
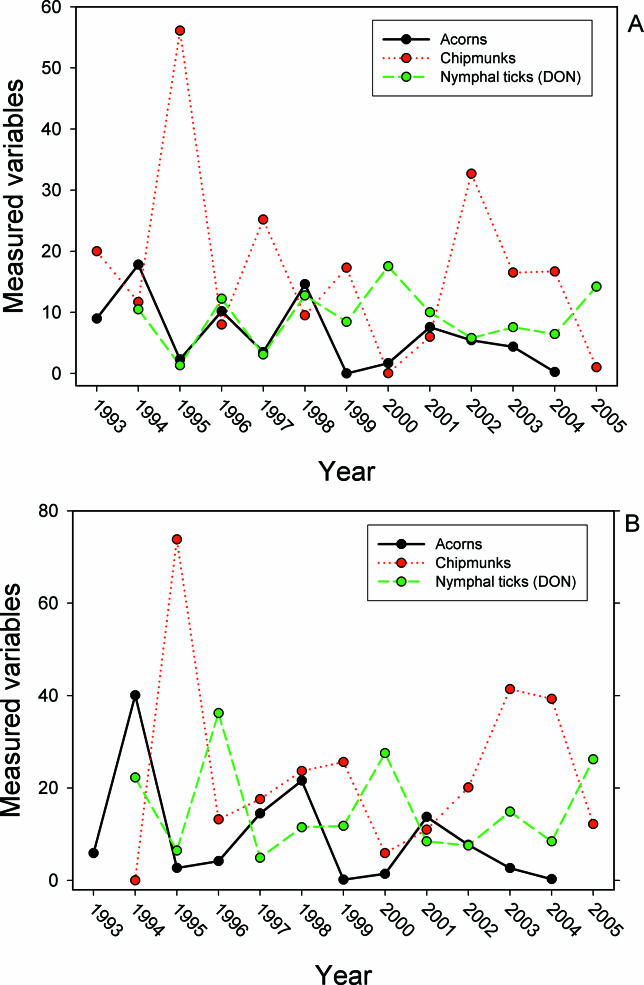
Time Series of Acorn, Tick, and Chipmunk Densities on Study Plots Shows time series of acorn density (acorns per square meter), chipmunk density (number per 2.25-ha grid), and DON (number per 100 m ^2^) on the two longest-established study plots, Henry Farm (A) and Teahouse (B). Note that, typically, chipmunk density tracks acorn density with a 1-y lag, and DON tracks chipmunk density also with a 1-y lag.

Densities of both mice and chipmunks responded strongly to the prior year's acorn abundance, and in both cases the relationship was best described by saturating power functions ( Figure 5A and 5B). As a result of their similar responses to acorn abundance, the abundance of mice and chipmunks was strongly correlated among years ( *r* = 0.62). A linear model of the mouse–chipmunk correlation was superior to any of the nonlinear models, with a slope of 0.25 indicating that mice are consistently about four times as abundant as chipmunks ( Figure 5C). As a consequence of acorns influencing rodent abundances, and rodents influencing nymphal abundances, we observed a general pattern in which DON tracked rodents, and rodents tracked acorns, each effect displaying a 1-y lag ( Figure 6).

Given their relatively long generation times and low reproductive potential, it is unrealistic to expect deer population abundance to track annual variation in acorn production. However, the potential exists for deer to be attracted from nonoak to oak habitats by the presence of abundant acorns [ 39, 42]. Because deer are an important host for adult ticks, and adult ticks lay eggs where they drop off their hosts, we expected that DOL in any given year would reflect deer space use the prior fall and should be positively correlated with acorn abundance. We found a weak, linear relationship between deer *_t_*_−2_ and DOL *_t_*_−1_, with this model being only a slight improvement over the means model (ΔAIC _corr_ = 0.39; *R* ^2^ = 0.04). In addition, no relationship existed between acorns _*t*−2_ and DOL *_t_*_−1_; all models, whether linear or nonlinear, were worse than the means model.

## Discussion

Climate, deer, and acorns each have been proposed as primary determinants of temporal variation in risk of human exposure to Lyme disease, as measured by abundance and *Borrelia*-infection prevalence in nymphal *Ixodes* ticks. Using a model comparison approach and a 13-y dataset, we found weak support for climate variables, no support for deer, and strong support for an effect of acorns, mediated by acorn effects on white-footed mice and eastern chipmunks, which host many larval ticks and are competent reservoirs for *B. burgdorferi.*

### Climate Variables

Of the four climate variables, two influenced DON and DIN, but they did so in unanticipated ways. Both DON and DIN increased linearly, albeit weakly, with increases in the prior year's temperature (GDD *_t_*_−1_). This result conflicts with the expectation that heat-caused mortality is an important regulator of tick abundance [ 15, 43], but is consistent with the finding that (detrended) incidence of Lyme disease in people is positively correlated with summer temperatures in the prior year [ 44]. Both DON and DIN were influenced, again weakly, by precipitation in the current but not prior year, but intermediate levels of precipitation favored highest nymphal abundances. Again, this result conflicts with the expectation that tick survival increases linearly with ambient moisture [ 18]. Low DON and DIN in years of high precipitation could be caused by either flood-induced mortality or that caused by natural enemies (e.g., fungi) that are facilitated by high moisture [ 45]. As expected, none of the climate variables influenced NIP. NIP is determined by the proportions of larval tick meals taken from the various host species, which vary strongly in their reservoir competence. It seems unlikely that climate variables will influence the choice of hosts by larval ticks or relative abundances of different hosts. It remains possible that climate variables other than the ones we examined influence stage-specific tick survival and abundance and that the weak or absent effects we observed are a result of not including more important variables. Our analytical approach could easily support assessments of other variables for which there is some a priori expectation of an effect.

### Deer Variables

The assertion that variable deer abundance is responsible for variable abundance of blacklegged ticks and hence Lyme-disease risk has become almost axiomatic [ 46, 47]. This seems to be based largely on repeated observations that removal of deer by fencing or shooting causes dramatic declines in tick abundance. However, some evidence suggests that the effects of deer on ticks are nonlinear, weak, and variable with tick life stage [ 26– 28]. An effect of deer abundance in year *t*−2 on nymphs in year *t* would be expected if (1) the number of autumn blood meals taken by adult ticks is correlated with the abundance of deer, causing (2) density of larval ticks in year *t*−1 to be correlated with deer in year *t*−2, and if (3) abundance of nymphs in year *t* is correlated with that of larvae in year *t*−1. Here, we found that more than 3-fold variation in our indices of deer abundance did not affect subsequent nymph abundance. This observation supports the assertion that, once deer abundance exceeds a low threshold value, further increases in deer density have little if any effect on nymphal densities [ 48]. We did observe a weak effect of deer on subsequent larval abundance, but larval abundance in any given year had no impact on next year's abundance of nymphs. This clear but surprising result indicates a decoupling of stage-specific abundances and suggests instead that abundance of nymphs depends on larval feeding opportunities the prior year (see below). The lack of demographic forcing from larval to nymphal stage each year also suggests that the effect of larval host abundance in any given year should penetrate only to the next year and not beyond.

### Acorn and Rodent Variables

Previous research [ 32, 40] supported an effect of acorns in year *t*−2 on nymphs in year *t* via two pathways, one in which abundant acorns *_t_*_−2_ boosted larvae *_t_*_−1_ by enhancing deer *_t_*_−2_, and the other in which acorns _*t*−2_ boosted rodents *_t_*_−1_, which in turn elevated nymphs *_t_* ( Figure 1). Our results strongly support the second, rodent-driven pathway ( Figure 6) and refute the first, deer-driven one. Although univariate models with GDD *_t_*_−1_, PPT *_t_*, acorns *_t_*_−2_, mice *_t_*_−1_, and total rodents *_t_*_−1_ all had support, the best model had chipmunks in the previous year as the sole predictor of DON. A stronger role for chipmunks than for mice was somewhat surprising given the lower population densities of chipmunks ( Figure 4) and only modestly higher average larval burdens on chipmunks [ 33] at our sites. However, less intense grooming of larval ticks by chipmunks [ 49] could facilitate larval survival to the nymphal stage. Elsewhere in the North American range of Lyme disease, chipmunks have been postulated to play a critical role in the enzootic cycle [ 34, 35, 50].

In contrast to previous results based on a shorter time series [ 41] and to expectations based on the high reservoir competence of mice and chipmunks [ 33], neither mice *_t_*_−1_ nor chipmunks *_t_*_−1_ influenced NIP. Instead, we found that the univariate model of acorns _*t*−2_ was the only one supported by the data. Empirically based models [ 51] indicate that NIP varies strongly with variable species composition in the community of hosts for larval ticks. Consequently, an impact of acorns but not of single host species would be expected if acorns alter the species composition in the host community. In addition to mice and chipmunks, acorns are likely to influence abundance and space use by gray squirrels *(Sciurus carolinensis),* raccoons *(Procyon lotor),* and turkeys *(Meleagris gallopavo).* The former two species are incompetent reservoirs [ 33], and the latter is unlikely to be a competent reservoir [ 52]. Therefore, by influencing a suite of hosts that both stimulate and depress NIP, acorns might be expected to explain more of the variation in NIP than would any single host species.

Because DIN is the product of DON and NIP, one might expect that the best explanatory model would be more complex than those for its component parts. Indeed, we found that the model of multiplicative effects of mice *_t_*_−1_ (the second best predictor of DON) and acorns _*t*−2_ (the best predictor of NIP) had the most support. The model with multiplicative effects of chipmunks *_t_*_−1_ (the best predictor of DON) and acorns _*t*−2_ was almost as strongly supported.

Tick abundance and Lyme-disease risk can be high in habitats with few or no oaks [ 21]; therefore, we do not expect that acorn abundance will be a universal predictor of risk [ 44]. However, small rodent hosts are almost always involved in risk as both permissive tick hosts and competent *Borrelia* reservoirs. Population densities of mice and chipmunks vary dramatically among years in both oak-dominated and nonoak-dominated forests [ 53], and we expect that the increase in Lyme-disease risk that accompanies high rodent densities should be widespread, no matter the causes of rodent fluctuations. Acorns provide a convenient leading indicator of rodent abundance, and seeds of other tree species [ 53] or predators [ 54, 55] might act similarly.

Previous studies of the determinants of variable Lyme-disease risk or incidence have tended to focus on one or a small number of potential independent variables, and statistically significant effects of both climate [ 16– 18] and deer [ 23– 25] have been described. When we included candidate climate variables, deer indices, and larval tick density together with densities of mice, chipmunks, and acorns, our model comparison methods never selected models incorporating climate, deer, or larvae and always selected models with either rodents, acorns, or both, as explanatory variables. Effects of variable climate or deer abundance on Lyme-disease risk might achieve statistical significance without being biologically important if statistical models fail to assess more potent independent variables. It remains possible that variable climate and deer abundance affect large-scale spatial variation in Lyme-disease risk even if their impacts on temporal variability are weak. Our long-term studies of Lyme-disease risk at the epicenter of the epidemic in North America strongly implicate a role for population density of rodent hosts and their food resources. We suggest that masting indices could be used to alert the public when years of high Lyme-disease risk are anticipated.

## Materials and Methods

### Study sites.

Field studies were conducted on the property of the Institute of Ecosystem Studies (IES) in Dutchess County, southeastern New York (lat 41 ^°^50′N, long 73 ^°^45′W), in the center of the northeastern US endemic zone for Lyme disease. Dutchess County has had among the highest incidence rates of Lyme disease in the US during the past 10 y [ 56]. IES forests are typical of the eastern deciduous forests of New York and New England, dominated by oaks *(Quercus rubra* and *Quercus prinus)* in the overstory (57%–70% oak relative basal area; [ 42]), with oak and sugar maple *(Acer saccharum)* seedlings, maple-leaved viburnum *(Viburnum acerifolium),* witch hazel *(Hamamelis virginiana),* and ironwood *(Ostrya virginiana)* common in the understory. Two 2.25-ha plots (150 m × 150 m) were established in 1991, and four more were added in 1995 to comprise three pairs of plots with ca. 150 m separating members of a pair and more than 700 m separating pairs.

### Acorn sampling.

Acorn abundance was monitored on each of the six plots by placing circular baskets under the canopies of mature oaks distributed throughout the plot. The original two plots had twenty 0.5-m ^2^ baskets, and the remaining four had twenty-five 1.0-m ^2^ baskets. Seed baskets were supported by monofilament line attached to nylon stakes and were resistant to seed predators. Intact, mature acorns were counted monthly during autumn of each year, and the total number of acorns from all baskets within a plot was divided by the total basket area to derive an estimate of annual acorn production on each plot. Full-season acorn data were collected from 1993 through 2004 on the original two plots and from 1999 through 2004 on the remaining four plots. Acorn density in year *t*−2 was used as an independent variable potentially affecting Lyme-disease risk factors.

### Small-mammal sampling.

Each year from 1991 (the two original plots) or from 1995 (remaining plots) through 2005 we have monitored abundance of small mammals at IES using capture–mark–recapture techniques. On each plot we established an 11 × 11 point grid of Sherman live traps, with 15 m between trap stations and two traps per station, for a total of 242 traps per grid. Trapping was conducted for 2–3 consecutive days every 3–4 wk, generally from May to November of each year. Traps were baited with crimped oats (with sunflower seeds and cotton batting added during cold weather), set at 1600 hours and checked between 0800 hours and about 1200 hours the following morning. This schedule allowed us to capture both diurnal (chipmunks) and nocturnal (mice) small mammals. These two species comprised more than 90% of captures. Small mammals were marked with individually numbered metal ear tags and released after handling at the point of capture. Data on age, sex, body mass, ectoparasite burden, and trap station were recorded on each capture. Protocols for animal handling were approved annually by an Institutional Animal Care and Use Committee.

We estimated population densities of white-footed mice and eastern chipmunks by inputting data from all trap sessions in a year into the Jolly-Seber open population model in program POPAN5 [ 57]. We selected the Jolly-Seber model that incorporates individual heterogeneity in capture probability. Because we were interested in assessing the importance of rodent abundance on nymphal tick abundance the following year, we focused on estimating rodent densities in midsummer, which is the time of peak activity of larval I. scapularis at our sites [ 10]. In order to create a standard metric when actual trapping sessions varied in time, we estimated mouse and chipmunk abundance for each grid and year on August 15 by linear interpolation between rodent abundance estimates for trap sessions immediately before and after August 15. Rodent densities in year *t*−1 were used as independent variables potentially affecting Lyme-disease risk factors.

### Deer abundance estimates.

Deer abundance was estimated at two levels, one at the scale of the IES property and the other at the scale of individual plots. For estimating property-wide deer abundance annually, we used population estimates from the IES limited-access bow-hunting program, which has run continuously from 1987 to the present [ 58]. Between seven and 11 hunters per year hunted an average of 40 h each (range, 29–54 h) between mid-October and mid-November. All hunters were IES staff members or volunteers working with the staff wildlife biologist. Each was given exclusive access to one of 34 discrete hunting areas averaging 22 ha (range, 9.3–35.7 ha). Virtually all hunting was from tree stands. Each day, hunters reported the number of hours hunted and the number of deer sighted, and these data were converted to deer observed per hour hunted. As a validation of this method, we asked whether deer observed per hour by bow hunters were correlated with deer counts from annual autumn spotlighting surveys conducted from 1987 to 2000 (details in [ 58]) and found that the two census methods were highly correlated ( *r* = 0.70, *df* = 11, *p* = 0.01; [ 58]).

To estimate deer distribution on a smaller scale, we used deer browse surveys conducted each spring (1983–2004) at a number of sites (range, 38–50) distributed throughout the IES property. Commonly browsed tree species or genera [ 59, 60] have served as an index to trends in browsing rates. These species/genera include red maple *(Acer rubrum),* sugar maple *(A. saccharum),* serviceberry *(Amelanchier arborea),* black birch *(Betula lenta),* black cherry *(Prunus serotina),* oaks ( Quercus spp.), hickories ( Carya spp.), and ashes ( Fraxinus spp.). Browsed woody stems detected in spring reflect the distribution of deer foraging activity during the previous autumn and winter. Data were collected along transects of unrestricted width spaced either 200 m or 300 m apart. Starting points were associated with corners of a 100-m grid overlay of the property. A major compass direction was randomly drawn each year for transect direction. As index species were sighted, all buds below 2 m were counted and examined for browsing by deer. At least 100 buds in total were examined for each site. The percentage of available stems browsed was calculated for each site and for each index species. We selected browse survey plots closest to each of the trapping grids (less than 100 m distant) to estimate deer activity specific to each of the plots. Deer browsing intensity in year *t*−2 was used as an independent variable potentially affecting Lyme-disease risk factors.

### Climate variables.

An almost infinite number of climate variables (temperature, precipitation, minimum, maximum, variance, mean, specific to months or seasons, etc.) potentially could influence tick survival and densities of nymphs. Consequently, the probability of uncovering spurious relationships between climate and tick abundance is quite high if many explanatory variables are included without clear a priori justification [ 61]. To avoid this problem, we selected climate variables that have been reported to influence either changes in the abundance of immature blacklegged ticks or in human Lyme-disease incidence [ 16, 17]. The climate variables selected represent temperature and precipitation conditions in either the current year (year *t*) or the prior year (year *t*−1). Values for year *t* reflect potential impacts of climate on survival of the current year's nymphal stage, whereas those for year *t*−1 represent possible effects of climate on survival of the prior year's larval stage. Following Jones and Kitron [ 16], for temperature data, we included only those months from the beginning of the growing season through the end of the activity season for the appropriate life stage (July for nymphs, September for larvae; [ 16, 41]). Specifically, we used cumulative growing degree days (GDD) for March 1–June 30 of year *t,* and cumulative GDD for March 1–September 30 of year *t*−1. For precipitation, we included only those months from the onset of potential soil moisture limitation (May) to the end of the appropriate activity period [ 16]; i.e., PPT (in mm) for May 1–June 30 of year *t;* and PPT for May 1–September 30 of year *t*−1 [ 16]. All climate data came from the IES environmental monitoring station ( http://www.ecostudies.org/emp_purp.html, located less than 1.5 km from our field plots

### Tick and *Borrelia* sampling.

Estimates of the abundance and infection prevalence of nymphal ticks comprised the response variables of interest. In addition, estimates of larval abundance in year *t*−1 were used as an independent variable. We monitored the abundance of larval and nymphal ticks in each plot and year by dragging 1-m ^2^ white corduroy drag cloths [ 62] along 450 m of transects approximately every 3 wk from April through November. Drag cloths were examined and all ticks counted and removed every 30 m. Frequent sampling in the early 1990s revealed that peak host-seeking activity for larvae occurred in mid- to late August, and for nymphs in mid- to late June, and we timed our annual sampling to coincide with these peaks. For each plot and year, we estimated larval and nymphal abundance as the peak density (ticks per 100 m ^2^). Peak densities were highly correlated with cumulative seasonal densities (correlation coefficients typically greater than 0.80; R. Ostfeld, unpublished data) on each plot.

Infection of individual ticks with B. burgdorferi was determined using direct immunofluorescence assay (DIA). Ticks were washed once in 70% ethanol and twice in deionized water and ground in phosphate-buffered saline (PBS). Three 5-ml aliquots of tick suspension were placed in separate wells in a multiwell slide, air-dried, and fixed in cold acetone for 10 min. Fluorescein rabbit anti- B. burgdorferi conjugate was incubated in wells at 37 °C for 45 min, after which slides were washed in PBS, dried, and mounted with a coverslip. Slides were examined systematically to categorize each tick as either infected or uninfected. On average, 378 nymphs (range, 146–660) were examined for infection each year. Our estimates of tick infection based on DIA have been verified by virtually identical infection prevalence estimates (within 1.6 percentage points) for IES ticks based on PCR and reverse-line blotting [ 63, 64].

### Statistical methods.

Our goal was to evaluate the strength of evidence for effects of a series of plausible independent variables on temporal variation in entomological risk of human exposure to Lyme disease ( Figure 1). Risk was measured by annual estimates at individual sample locations for the DON, NIP, and their product, the DIN. For each of the three response variables (DON, NIP, DIN) we compared the strength of evidence for a series of alternative, competing regression models using AIC _corr_. We created both linear and nonlinear (exponential, power function, Gaussian) models, as appropriate. We first compared evidence for each of the 11 independent variables separately by comparing the AIC _corr_ of their regression models to the AIC _corr_ value of an intercept-only (i.e., mean) model. The 11 independent variables included two temperature variables, two precipitation variables, two indices of deer abundance, abundance of larval ticks, acorn abundance, and three measures of small mammal abundance. We checked for colinearity among the independent variables using correlation coefficients. Because there were missing values for some variables in some years and plots, the univariate models were compared against mean models estimated with the same subset of (nonmissing) observations. We then tested series of increasingly complex models by combining sets of independent variables for which there was evidence (as measured by AIC) of univariate effects. Our choices for the forms of the multiple regression models were guided by the dictates of parsimony: while our dataset represents an enormous sampling effort over a 13-y period, the actual sample sizes (a given plot in a given year) were still relatively small, ranging from 42 to 58 observations for the various models.

We used simulated annealing (a global optimization algorithm) to find the maximum likelihood estimates for the parameters of each model. The observations were assumed to be normally distributed with a homogeneous variance. Examination of the residuals indicates that this assumption was appropriate for all three of the response variables. The specific annealing algorithm we used (based on Goffe et al. [ 65]) allows a bounded search in cases where a range of parameter values is either mathematically inconsistent or biologically unreasonable (i.e., negative densities or variance estimates). The sample locations were widely enough distributed that we are confident in assuming that the error terms in our models are spatially independent, and there was no evidence of temporal autocorrelation in the residuals. The simulated annealing and all of the statistical analyses were done using R ( http://www.r-project.org).

## Abbreviations

AIC _corr_: Akaike's information criterion
DIN: density of infected nymphs
DOL: density of larvae
(DOL _*t*−1 _): prior year's density of larvae
DON: density of nymphs
GDD: growing degree days
GDD _*t*−1 _: growing degree days in the previous year
IES: Institute of Ecosystem Studies
NIP: nymphal infection prevalence
PPT: total precipitation
PPT _*t*_: total growing season precipitation in the current year

## References

[pbio-0040145-b001] Antonovics J, Iwasa Y, Hassell M (1995). A generalized model of parasitoid, venereal, and vector-based transmission processes. Am Nat.

[pbio-0040145-b002] Rogers D, Randolph S, Snow R, Hay S (2002). Satellite imagery in the study and forecast of malaria. Nature.

[pbio-0040145-b003] Gubler DJ, Reiter P, Ebi KL, Yap W, Nasci R (2001). Climate variability and change in the United States: Potential impacts on vector- and rodent-borne diseases. Environ Health Perspect.

[pbio-0040145-b004] Harvell CD, Mitchell CE, Ward JR, Altizer S, Dobson A (2002). Climate warming and disease risks for terrestrial and marine biota. Science.

[pbio-0040145-b005] Hubalek Z (2005). North Atlantic weather oscillation and human infectious diseases in the Czech Republic, 1951–2003. Eur J Epidemiol.

[pbio-0040145-b006] Centers for Disease Control and Prevention [CDC] (2002). Lyme disease—United States, 2000. MMWR Morb Mortal Wkly Rep.

[pbio-0040145-b007] Centers for Disease Control and Prevention [CDC] (2006). Reported cases of Lyme disease—United States, 2004.

[pbio-0040145-b008] Keirans JE, Hutcheson HJ, Durden LA, Klompen JSH (1996). *Ixodes (Ixodes) scapularis* (Acari: Ixodidae): Redescription of all active stages, distribution, hosts, geographical variation, and medical and veterinary importance. J Med Entomol.

[pbio-0040145-b009] Barbour AG, Fish D (1993). The biological and social phenomenon of Lyme disease. Science.

[pbio-0040145-b010] Ostfeld RS (1997). The ecology of Lyme-disease risk. Am Sci.

[pbio-0040145-b011] Ostfeld RS, Keesing F, Schauber EM, Schmidt KA, Aguirre A, Ostfeld RS, Tabor G, House CA, Pearl M (2002). The ecological context of infectious disease: Diversity, habitat fragmentation, and Lyme disease risk in North America. Conservation medicine: Ecological health in practice.

[pbio-0040145-b012] Bertrand MR, Wilson ML (1996). Microclimate-dependent survival of unfed adult
Ixodes scapularis (Acari: Ixodidae) in nature: Life cycle and study design implications. J Med Entomol.

[pbio-0040145-b013] Brownstein JS, Holford TR, Fish D (2005). Effect of climate change on Lyme disease risk in North America. EcoHealth.

[pbio-0040145-b014] Ashley ST, Meentemeyer V (2004). Climatic analysis of Lyme disease in the United States. Clim Res.

[pbio-0040145-b015] Needham GR, Teel PD (1991). Off-host physiology of Ixodid ticks. Annu Rev Entomol.

[pbio-0040145-b016] Jones CJ, Kitron UD (2000). Populations of
Ixodes scapularis (Acari: Ixodidae) are modulated by drought at a Lyme disease focus in Illinois. J Med Entomol.

[pbio-0040145-b017] Subak S (2003). Effects of climate on variability in Lyme disease incidence in the Northeastern United States. Am J Epidemiol.

[pbio-0040145-b018] McCabe GJ, Bunnell JE (2004). Precipitation and the occurrence of Lyme disease in the northeastern United States. Vector Borne Zoonotic Dis.

[pbio-0040145-b019] Lane RS, Piesman J, Burgdorfer W (1991). Lyme borreliosis: Relation of its causative agents to its vector and hosts in North America and Europe. Annu Rev Entomol.

[pbio-0040145-b020] Daniels TJ, Fish D, Schwartz I (1993). Reduced abundance of
Ixodes scapularis (Acari: Ixodidae) and Lyme disease risk by deer exclusion. J Med Entomol.

[pbio-0040145-b021] Ginsberg HS, Zhioua E, Mitra S, Fischer J, Buckley PA (2004). Woodland type and spatial distribution of nymphal
Ixodes scapularis (Acari: Ixodidae). Environ Entomol.

[pbio-0040145-b022] Rand PW, Lubelczyk C, Holman MS, Lacombe EH, Smith RP (2004). Abundance of
Ixodes scapularis (Acari: Ixodidae) after the complete removal of deer from an isolated offshore island, endemic for Lyme disease. J Med Entomol.

[pbio-0040145-b023] Wilson ML (1998). Distribution and abundance of
Ixodes scapularis (Acari: Ixodidae) in North America: Ecological processes and spatial analysis. J Med Entomol.

[pbio-0040145-b024] Stafford KC, Denicola AJ, Kilpatrick HJ (2003). Reduced abundance of
Ixodes scapularis (Acari: Ixodidae) and the tick parasitoid
Ixodiphagus hookeri (Hymenoptera: Encyrtidae) with reduction of white-tailed deer. J Med Entomol.

[pbio-0040145-b025] Rand PW, Lubelczyk C, Lavigne GR, Elias S, Holman MS (2003). Deer density and the abundance of
Ixodes scapularis (Acari: Ixodidae). J Med Entomol.

[pbio-0040145-b026] Jordan RA, Schulze TL (2005). Deer browsing and the distribution of
Ixodes scapularis (Acari: Ixodidae) in central New Jersey forests. Environ Entomol.

[pbio-0040145-b027] Lubelczyk CB, Elias SP, Rand PW, Holman MS, Lacombe EH (2004). Habitat associations of
Ixodes scapularis (Acari: Ixodidae) in Maine. Environ Entomol.

[pbio-0040145-b028] Schultze TL, Jordan RA, Hung RW (2001). Potential effects of animal activity on the spatial distribution of
Ixodes scapularis and
Amblyomma americanum (Acari: Ixodidae). Environ Entomol.

[pbio-0040145-b029] Donahue JG, Piesman J, Spielman A (1987). Reservoir competence of white-footed mice for Lyme disease spirochetes. Am J Trop Med Hyg.

[pbio-0040145-b030] Mather TN, Wilson ML, Moore SI, Ribeiro JMC, Spielman A (1989). Comparing the relative potential of rodents as reservoirs of the Lyme disease spirochete
*(Borrelia burgdorferi)*. Am J Epidemiol.

[pbio-0040145-b031] Mather TN, Ginsberg HS (1993). The dynamics of spirochete transmission between ticks and vertebrates. Ecology and environmental management of Lyme disease.

[pbio-0040145-b032] Ostfeld RS, Jones CG, Wolff JO (1996). Of mice and mast: Ecological connections in eastern deciduous forests. Bioscience.

[pbio-0040145-b033] LoGiudice K, Ostfeld RS, Schmidt KA, Keesing F (2003). The ecology of infectious disease: Effects of host diversity and community composition on Lyme disease risk. Proc Natl Acad Sci U S A.

[pbio-0040145-b034] Mannelli A, Kitron U, Jones CJ, Slajchert TL (1993). Role of the eastern chipmunk as a host for immature
Ixodes dammini (Acari: Ixodidae) in northwestern Illinois. J Med Entomol.

[pbio-0040145-b035] Slajchert T, Kitron UD, Jones CJ, Mannelli A (1997). Role of the eastern chipmunk
*(Tamias striatus)* in the epizootiology of Lyme borreliosis in northwestern Illinois, USA. J Wildl Dis.

[pbio-0040145-b036] Elkinton JS, Healy WM, Buonaccorsi JP, Boettner GH, Hazzard AM (1996). Interactions among gypsy moths, white-footed mice, and acorns. Ecology.

[pbio-0040145-b037] Wolff JO (1996). Population fluctuations of mast-eating rodents are correlated with production of acorns. J Mammal.

[pbio-0040145-b038] McShea WJ (2000). The influence of acorn crops in annual variation in rodent and bird populations. Ecology.

[pbio-0040145-b039] McShea WJ, Schwede G (1993). Variable acorn crops: Responses of white-tailed deer and other mast consumers. J Mammal.

[pbio-0040145-b040] Jones CG, Ostfeld RS, Richard MP, Schauber EM, Wolff JO (1998). Chain reactions linking acorns to gypsy moth outbreaks and Lyme disease risk. Science.

[pbio-0040145-b041] Ostfeld RS, Schauber EM, Canham CD, Keesing F, Jones CG (2001). Effects of acorn production and mouse abundance on abundance and
Borrelia burgdorferi infection prevalence of nymphal
Ixodes scapularis ticks. Vector Borne Zoonotic Dis.

[pbio-0040145-b042] Ostfeld RS, Keesing F, Jones CG, Canham CD, Lovett GM (1998). Integrative ecology and the dynamics of species in oak forests. Integr Biol.

[pbio-0040145-b043] Mount GA, Haile DG, Daniels E (1997). Simulation of blacklegged tick (Acari: Ixodidae) population dynamics and transmission of
Borrelia burgdorferi. J Med Entomol.

[pbio-0040145-b044] Schauber EM, Ostfeld RS, Evans A (2005). What is the best predictor of annual Lyme disease incidence: Weather, mice, or acorns?. Ecol Appl.

[pbio-0040145-b045] Benjamin MA, Zhioua E, Ostfeld RS (2002). Benjamin MA, Zhioua E, Ostfeld RS (2002) Laboratory and field evaluation of the entomopathogenic fungus Metarhizium anisopliae (Deuteromycetes) for controlling questing adult Ixodes scapularis (Acari: Ixodidae). J Med Entomol.

[pbio-0040145-b046] Barbour AG (1998). Fall and rise of Lyme disease and other
*Ixodes* tick-borne infections in North America and Europe. Br Med J.

[pbio-0040145-b047] Spielman A (1994). The emergence of Lyme disease and human babesiosis in a changing environment. Ann N Y Acad Sci.

[pbio-0040145-b048] Van Buskirk J, Ostfeld RS (1995). Controlling Lyme disease by modifying density and species composition of tick hosts. Ecol Appl.

[pbio-0040145-b049] Shaw MT, Keesing F, McGrail R, Ostfeld RS (2003). Factors influencing the distribution of larval blacklegged ticks on rodent hosts. Am J Trop Med Hyg.

[pbio-0040145-b050] Schulze TL, Jordan RA, Schulze CJ (2005). Host associations of
Ixodes scapularis (Acari: Ixodidae) in residential and natural settings in a Lyme disease-endemic area in New Jersey. J Med Entomol.

[pbio-0040145-b051] Ostfeld RS, LoGiudice K (2003). Community disassembly, biodiversity loss, and the erosion of an ecosystem service. Ecology.

[pbio-0040145-b052] Ostfeld RS, Lewis DN (1999). Experimental studies of interactions between wild turkeys and blacklegged ticks. J Vector Ecol.

[pbio-0040145-b053] McCracken KE, Witham JW, Hunter ML (1999). Relationships between seed fall of three tree species and
Peromyscus leucopus and
Clethrionomys gapperi during 10 years in an oak-pine forest. J Mammal.

[pbio-0040145-b054] Elias SP, Witham JW, Hunter ML (2004). Peromyscus leucopus abundance and acorn mast: Population fluctuation patterns over 20 years. J Mammal.

[pbio-0040145-b055] Ostfeld RS, Holt RD (2004). Are predators good for your health? Evaluating evidence for top–down regulation of zoonotic disease reservoirs. Front Ecol Environ.

[pbio-0040145-b056] Chow CC, Evans AS, Noonan-Toly CM, White D, Johnson GS (2003). Lyme disease trends—Dutchess County, New York, 1992–2000. Mt Sinai J Med.

[pbio-0040145-b057] Arnason AN, Schwartz CJ (1999). Using POPAN-5 to analyze banding data. Bird Study.

[pbio-0040145-b058] Winchcombe RJ, Ostfeld RS (2001). Bowhunter observations versus spotlighting as an index to deer abundance. Northeast Wildl.

[pbio-0040145-b059] Bramble WC, Goddard MK (1953). Seasonal browsing of woody plants by white-tailed deer in the ridge and valley section of central Pennsylvania. J Forest.

[pbio-0040145-b060] Healy WM (1971). Forage preferences of tame deer in a northwest Pennsylvania clear-cutting. J Wildl Manage.

[pbio-0040145-b061] Anderson DR, Burnham KP, Thompson WL (2000). Null hypothesis testing: Problems, prevalence, and an alternative. J Wildl Manage.

[pbio-0040145-b062] Falco RC, Fish D (1992). A comparison of methods for sampling the deer tick,
*Ixodes dammini* in a Lyme disease endemic area. Exp Appl Acarol.

[pbio-0040145-b063] Qiu WG, Dykhuizen DE, Acosta MS, Luft BJ (2002). Geographic uniformity of the Lyme disease spirochete
*(Borrelia burgdorferi)* and its shared history with tick vector
*(Ixodes scapularis)* in the northeastern United States. Genetics.

[pbio-0040145-b064] Brisson D, Dykhuizen DE (2004). ospC diversity in
*Borrelia burgdorferi* Different hosts are different niches. Genetics.

[pbio-0040145-b065] Goffe WL, Ferrier GD, Rogers J (1994). Global optimization of statistical functions with simulated annealing. J Econom.

